# Association of nutrient-derived dietary patterns with sarcopenia and its components in community-dwelling older Japanese: a cross-sectional study

**DOI:** 10.1186/s12937-021-00665-w

**Published:** 2021-01-18

**Authors:** Yuri Yokoyama, Akihiko Kitamura, Satoshi Seino, Hunkyung Kim, Shuichi Obuchi, Hisashi Kawai, Hirohiko Hirano, Yutaka Watanabe, Keiko Motokawa, Miki Narita, Shoji Shinkai

**Affiliations:** 1 Research Team for Social Participation and Community Health, Tokyo Metropolitan Institute of Gerontology, 35-2 Sakae-cho, Itabashi-ku, Tokyo, 173-0015 Japan; 2 Research Team for Promoting Independence and Mental Health, Tokyo Metropolitan Institute of Gerontology, 35-2 Sakae-cho, Itabashi-ku, Tokyo, 173-0015 Japan; 3 Research Team for Human Care, Tokyo Metropolitan Institute of Gerontology, 35-2 Sakae-cho, Itabashi-ku, Tokyo, 173-0015 Japan; 4 Gerodontology, Department of Oral Health Science, Faculty of Dental Medicine, Hokkaido University, Nishi-7, Kita-13, Kita-ku, Sapporo, Hokkaido 060-8586 Japan; 5 Tokyo Metropolitan Institute of Gerontology, 35-2 Sakae-cho, Itabashi-ku, Tokyo, 173-0015 Japan

**Keywords:** Sarcopenia, Dietary pattern, Dietary quality, Reduced rank regression

## Abstract

**Background:**

Diet is a modifiable factor affecting sarcopenia, and accumulating evidence links dietary factors to muscle mass, strength, and function in older adults. However, few studies have examined the association of dietary patterns with sarcopenia. This study examined the association of dietary patterns derived by reduced-rank regression (RRR) with sarcopenia and its components in community-dwelling older Japanese.

**Methods:**

We conducted a cross-sectional study of 1606 community-dwelling adults aged 65 years or older. Dietary intake was assessed by a validated, self-administered diet history questionnaire. Nutrient-derived dietary patterns were identified by using RRR, with sarcopenia-related nutrients (protein, vitamin D, vitamin C, vitamin E, folate, vitamin K, magnesium, iron, and calcium intakes) as response variables. Sarcopenia was defined by using the algorithm of the Asian Working Group for Sarcopenia 2019. Multivariate regression and logistic regression were used to examine the association of dietary patterns with sarcopenia and its components.

**Results:**

The first RRR dietary pattern was characterized by high intakes of fish, soybean products, potatoes, most vegetables, mushrooms, seaweeds, and fruit and a low intake of rice and was associated with decreased prevalence of sarcopenia: the multivariable-adjusted odds ratio of sarcopenia was 0.57 (95% confidence interval, 0.34–0.94; *p* for trend=0.022) in the highest versus the lowest tertile of dietary pattern. This dietary pattern was also significantly positively associated with usual gait speed (β: 0.02, *p*=0.024).

**Conclusions:**

A dietary pattern characterized by high intakes of fish, soybean products, potatoes, most vegetables, mushrooms, seaweeds, and fruits and low rice intake was inversely associated with sarcopenia in community-dwelling older Japanese.

**Supplementary Information:**

The online version contains supplementary material available at 10.1186/s12937-021-00665-w.

## Background

Sarcopenia—the loss of muscle mass and strength that occurs with advancing age—is associated with physical disability, poor quality of life, and increased mortality in older adults [1]. Because of the rapid ageing of the world’s population, identifying modifiable risk factors that prevent or delay sarcopenia onset is a public health priority.

Diet is a modifiable factor affecting sarcopenia, and a growing body of evidence links dietary factors to muscle mass, strength, and function in older adults [2–7]. In addition to evaluating the roles of single nutrients (protein and vitamin D) [8, 9] and foods (vegetables and fruits, dairy foods, and fish) [10–12], studies are increasingly investigating associations of adherence to overall dietary pattern with sarcopenia and related outcomes [6, 7]. To date, two main approaches have been used to identify dietary patterns [13, 14]: the a priori approach uses diet quality scores or indices based on dietary guidelines, while the a posteriori approach uses statistical techniques such as principal component analysis (PCA) and cluster analysis based on dietary intake reported by a population. However, because these methods of analyzing dietary pattern do not attempt to identify dietary disease-specific patterns, the patterns identified are not always optimal for explaining diet–disease associations [15].

To refine patterns and target them to a specific disease outcome, reduced-rank regression (RRR) has been increasingly applied as an additional method in nutritional epidemiology [16]. In contrast to other approaches, RRR can account for the total dietary intake of study participants and identify dietary patterns associated with the intake of nutrients known to be related to the outcome measure [16]. Because nutrition intervention studies that used a single-nutrient approach (e.g. protein alone or protein in combination with physical exercise) to investigate sarcopenia have not always been successful [17], RRR analysis of dietary patterns in relation to sarcopenia could yield new insights and preventive strategies for sarcopenia. Previous studies reported that older adults with sarcopenia had lower intakes of several vitamins and minerals [18, 19]; therefore, nutrients other than protein and vitamin D may have direct and indirect effects on sarcopenia through multiple pathways. However, to our knowledge, RRR has not been used in studies of sarcopenia. Therefore, the aim of this study was to examine the association of dietary patterns derived by reduced-rank regression (RRR) with sarcopenia and its components in community-dwelling older Japanese.

## Methods

This study is reported in accordance with the Strengthening the Reporting of Observational Studies in Epidemiology-nutritional epidemiology (STROBE-nut) checklist (Additional file 1) [20].

### Study design and participants

The present study analyzed cross-sectional data previously collected for the Hatoyama Cohort Study, Kusatsu Longitudinal Study, and Itabashi Cohort Study, which are population-based cohort studies of community-dwelling adults aged 65 years or older. Data were obtained from comprehensive health examinations conducted in the same manner. The details of the study designs and participants have been reported elsewhere [21, 22]. We used data from the year in which brief diet history questionnaires (BDHQ) were distributed, all of which were collected during the period from 2012 through 2014. These studies were approved by the relevant institutional review board, and written informed consent was obtained from all participants.

**Fig. 1:**
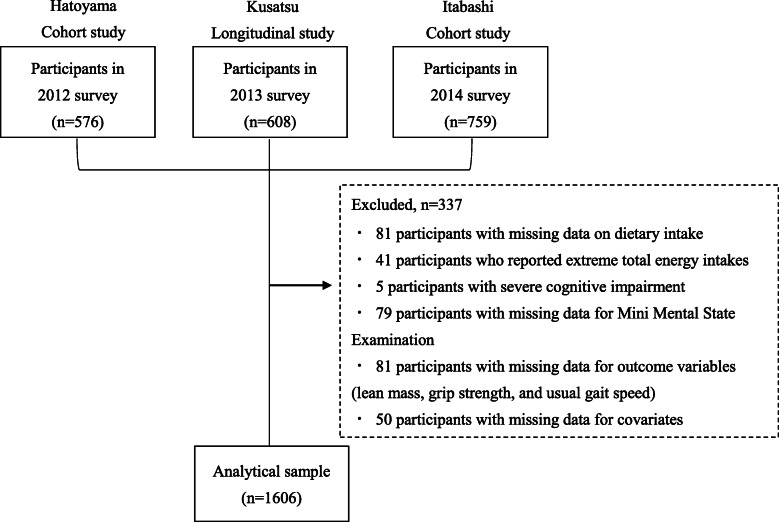
Flowchart for enrollment of study participants

Of the 1928 participants who agreed to participate in the study (*n*=576 in the Hatoyama Cohort Study; *n*=608 in the Kusatsu Longitudinal Study; *n*=759 in the Itabashi Cohort Study), we excluded those with missing information on dietary intake (*n* =81), those with under-reported and over-reported energy intakes (energy intakes less than half the requirement for the lowest physical activity category, according to the Dietary Reference Intakes for Japanese, 2015 [< 1050 kcal/day for men aged 65–69 years: *n*=1; < 925 kcal/day for men aged > 70 years: *n*=3; < 750 kcal/day for women aged > 70 years: *n*=2], or more than 1.5 times the energy requirement for the highest physical activity category [> 3750 kcal/day for men aged > 70 years: *n*=16; > 3000 kcal/day for women aged > 70 years: *n*=19]) [23, 24], those with severe cognitive impairment, defined as a Mini Mental State Examination Score (MMSE) of < 18 (*n*=5) [25], and those with missing data for the MMSE (*n*=79), the present outcome variables (*n*=81), or covariates (*n*=50). Ultimately, data from 1606 participants were analyzed (Fig. 1).

### Definition of sarcopenia

Sarcopenia was defined by using the algorithm of the Asian Working Group for Sarcopenia 2019 [26]. In accordance with the algorithm, we used the criteria low muscle mass, defined as an appendicular lean mass (ALM)/height^2^ of < 7.0 kg/m^2^ for men and < 5.7 kg/m^2^ for women; low muscle strength, defined as a grip strength of < 28 kg for men and < 18 kg for women; and low physical performance, defined as a gait speed of < 1.0 m/s for men and women. Participants with low muscle mass and either low muscle strength or low physical performance were categorized as having sarcopenia. The procedures for measuring muscle mass, grip strength, and gait speed have been described in detail previously [21, 27].

### Dietary assessment

Dietary habits during the preceding 1-month period were assessed with a validated brief self-administered diet history questionnaire (BDHQ) [28, 29]. The BDHQ is a four-page fixed-portion questionnaire and consists of five sections: 1) frequency of intake of 46 foods and non-alcoholic beverages; 2) daily frequency of rice and miso soup intake; 3) frequency of alcoholic drinking and amount of consumption for five alcoholic beverages per typical drinking occasion; 4) usual cooking methods; and 5) general dietary behavior. The validity of this questionnaire has been reported previously [28, 29]. To facilitate reading and completion for older adults, the present study used a large-print version that increased the number of pages to 10. Responses to the BDHQ were checked by research staff for completeness and, when necessary, were reviewed with participants to ensure answer clarity. Dietary intakes of 58 food and beverage items, energy, and specific nutrients were calculated by using an ad hoc computer algorithm based on the Standard Tables of Food Composition in Japan [30].

### Dietary pattern analysis

Dietary patterns were assessed by using RRR analysis, which identifies patterns in a set of food groups that explain as much variation as possible in response variables (e.g., nutrients or biomarkers) [16]. Unlike principal components analysis, which derives dietary patterns based on the covariance structure of foods, RRR allows us to more directly link the exploratory identification of dietary pattern to the outcome of interest by the choice of informative outcome-related response variables [31]. The RRR method has been described in detail elsewhere [16]. As predictors, 52 food and beverage items were energy-adjusted with the density method and used [32]. Response variables were selected for nine nutrients— protein, vitamin D, vitamin C, vitamin E, folate, vitamin K, magnesium, iron, and calcium—because these nutrients were reported to protect against sarcopenia or related outcomes in previous studies [5, 33–37] and were variables with a *P* value of < 0.2 [38] in a preliminary analysis of their intakes and sarcopenia in our study population. Although omega-3 polyunsaturated fatty acids (PUFAs) are of interest because of their demonstrated effects on skeletal muscle health [9], the *P* value for PUFAs was > 0.2 in our preliminary analysis. Therefore, we decided not to include PUFAs as a response variable. The extracted dietary pattern score was classified by tertile (T1–T3) in all participants.

### Covariates

The covariates used in the analyses were sex, age, study site, education, living arrangement (single, with spouse only, or other), smoking habit (never, former, or current smoker), drinking habit (never/rarely, sometimes, or every day), self-perceived chewing ability (can chew anything/almost anything, cannot chew much), frequency of going outdoors, self-reported medical history (hypertension, diabetes, heart disease, stroke, cancer, chronic obstructive pulmonary disease), body mass index (calculated as weight in kilograms divided by height in meters squared), and energy intake.

### Statistical analysis

Complete case analysis was used to address missing data (details of missing data are shown in Fig. 1). The characteristics of the study population, by category of dietary pattern score, were compared by using weighted one-way analysis of variance for continuous variables or the Mantel–Haenszel chi-square test for categorical variables. Associations between the dietary pattern extracted by RRR and nutrients as response variables were evaluated by using Spearman rank correlation coefficients. Multiple logistic regression and linear regression analyses were used to examine the associations of the first dietary pattern with sarcopenia and its components. In the logistic regression analysis, odds ratios (ORs) and 95% confidence intervals (CIs) were calculated for sarcopenia in relation to first dietary pattern scores, with the lowest tertile category defined as the reference. Tests for trend associations were based on assigning the ordinal numbers 0–3 to the three categories of first dietary pattern score. In the multiple linear regression analysis, we calculated mean (SE) values of ALM, grip strength, and usual gait speed in relation to tertile of dietary pattern. We also calculated the unstandardized partial coefficient, which reflects change in ALM, grip strength, and usual gait speed per one-tertile increase in conformity to the first dietary pattern.

The multivariate model was adjusted for the following potential confounding variables. The first model was adjusted for age (years, continuous), sex (men or women), and study site (Hatoyama, Kusatsu, or Itabashi) and further adjusted, in model 2, for education (years, continuous), living arrangement (single, with spouse only, or other), smoking habit (current, former, or never), drinking habit (every day, sometimes, or none/rarely), self-perceived chewing ability (can chew anything/almost anything or cannot chew much), frequency of going outdoors (more than once a day or less than once a day), medical history (hypertension, diabetes, cancer, stroke, heart disease, chronic obstructive pulmonary disease; yes or no), body mass index (kg/m^2^, continuous), total energy intake and MMSE score (score, continuous). These potential confounders were chosen after reviewing previous findings suggesting relations with both the exposure and outcome of interest.

A two-sided *P*-value of < 0.05 was considered to indicate statistical significance. Dietary pattern analyses (RRR) were performed with SAS version 9.4 (SAS Institute, Inc., Cary, NC, USA), and all other analyses were performed with IBM SPSS Statistics version 23 (IBM Corp, Armonk, NY, USA).

## Results

A total of 1606 participants were included in the present analyses. Table 1 shows explained variation in nutrients and food intake and correlations of nutrients (response variable) with RRR-derived dietary patterns. RRR extracted nine dietary patterns, which explained 25.0% of variation in food groups and 99.8% of variation in response variables. The first RRR pattern explained 7.53% of variation in food groups and 67. 51% of total variation in all responsible variables. The other dietary patterns explained less than 2.66 and 14.54% of the variation. Thus, only the first RRR pattern was considered in later analyses. The first RRR pattern highly correlated with intakes of each nutrient (Spearman rank correlation coefficient: ≥0.61 for all; *P*< 0.001).

**Table 1:** Explained variation in nutrients and food intake and Spearman rank correlations between nutrients (response variable) and RRR-derived dietary patterns

	Dietary patterns									Total explained variation
	1	2	3	4	5	6	7	8	9	
Explained variation in food intake, %	7.53	2.66	2.52	1.98	2.44	1.99	1.73	2.15	2.02	25.0
Explained variation in nutrients, %	67.51	14.54	6.19	4.36	3.09	1.69	1.24	0.76	0.46	99.8
Spearman rank correlation coefficient										
Protein	0.82*	0.45*	− 0.03	0.16*	0.02	−0.13*	− 0.16*	0.11*	0.08	
Vitamin D	0.61*	0.67*	0.08*	0.11*	−0.12*	0.17*	0.09*	0.00	−0.02	
Vitamin C	0.76*	−0.40*	0.38*	−0.18*	−0.14*	0.09*	−0.11*	0.09*	−0.04	
Vitamin E	0.79*	−0.11*	0.35*	0.25*	0.33*	0.01	0.00	−0.02	−0.01	
Folate	0.88*	−0.37*	0.03	−0.03	−0.15*	− 0.09*	0.09*	− 0.03	0.08*	
Vitamin K	0.73*	−0.37*	−0.46*	0.10*	0.09*	0.15*	−0.01	0.10*	−0.01	
Magnesium	0.94*	0.03	−0.13*	−0.09*	0.00	0.04	−0.20*	−0.16*	0.01	
Iron	0.93*	−0.11*	−0.09*	0.16*	−0.14*	− 0.17*	−0.04	0.01	−0.14*	
Calcium	0.80*	0.23*	−0.06	−0.42*	0.21*	−0.10*	0.06*	0.03	−0.03	

*P< 0.001

Factor loadings indicate the magnitude and direction of the contribution of a food group to a pattern. Table 2 shows factor loadings of food items in RRR-derived dietary pattern 1. A high positive loading shows a strong direct association between a food group and a pattern; a high negative loading indicates a strong inverse association. Foods with an absolute factor loading of 0.15 or greater were regarded as characteristic of the dietary pattern and included small fish with bones, dried fish/salted fish, oily fish, soybean products, potatoes, most vegetables, mushrooms, seaweeds, and persimmons/strawberries/kiwifruits; rice was the only food that had a factor loading less than − 0.15.

**Table 2:** Factor loadings of food items in RRR-derived dietary pattern 1

Food group	Factor loadings^a^
Low-fat milk and yogurt	0.07
Milk and yogurt	0.07
Chicken	0.07
Pork/beef	0.04
Ham/sausage/bacon	0.03
Liver	0.06
Squid/octopus/shrimp/shellfish	0.10
Small fish with bones	**0.26**
Canned tuna	0.08
Dried fish/salted fish	**0.16**
Oily fish	**0.15**
Lean fish	0.13
Egg	0.09
Tofu/*atsuage*	**0.22**
*Natto*	**0.19**
Potatoes	**0.17**
Pickled green leaf vegetables	0.06
Other pickles	0.06
Lettuce/cabbage (raw)	**0.21**
Green leaf vegetables	**0.32**
Cabbage/Chinese cabbage	**0.24**
Carrots/pumpkin	**0.25**
Japanese radishes/turnips	**0.21**
Other root vegetables	**0.23**
Tomatoes	**0.21**
Mushrooms	**0.23**
Seaweeds	**0.22**
Western-type confectioneries	−0.09
Japanese confectioneries	−0.03
Rice crackers/rice cakes/*okonomiyaki*	−0.05
Ice cream	−0.09
Citrus fruits	0.11
Persimmons/strawberries/kiwifruits	**0.15**
Other fruits	0.11
Mayonnaise/dressing	0.08
Bread	−0.05
Buckwheat noodles	−0.04
Japanese wheat noodles	−0.02
Chinese noodles	−0.10
Spaghetti and macaroni	−0.03
Green tea	0.11
Black tea/Oolong tea	0.02
Coffee	0.00
Cola drinks/soft drinks	−0.12
100% fruit and vegetable juices	0.06
Rice	**−0.19**
Miso soup	−0.03
*Sake*	−0.11
Beer	−0.11
*Shochu*	−0.13
Whisky	−0.09
Wine	−0.03

atsuage, deep-fried tofu; okonomiyaki, meat/fish and vegetables pancake; Shochu, a Japanese distilled beverage. ^a^Factor loadings with an absolute value of ≥0.15 are shown in bold

Table 3 shows the characteristics of the study participants, by tertile of dietary pattern 1 scores. Those with a higher dietary pattern score were more likely to be women and living alone, less likely to smoke and drink, and more likely to be able to chew most foods. In addition, they were less likely to have heart disease, had lower body mass index values and energy intakes, and had higher MMSE scores.

**Table 3:** Characteristics of study population, by tertile of dietary pattern 1 scores

	Dietary pattern 1 scores			p*
	Tertile 1 (low)	Tertile 2	Tertile 3 (high)	
No. of participants	535	536	535	
Age (y)	73.0 (5.8)	73.9 (5.7)	73.7 (5.5)	0.067
Education (y)	12.2 (3.0)	12.0 (2.9)	11.9 (2.8)	0.064
Women (%)	31.4	55.0	73.3	< 0.001
Study area				
Hatoyama	36.6	31.5	27.9	0.006
Kusatsu	27.3	30.4	30.7	
Itabashi	36.1	38.1	41.5	
Living alone (%)	18.9	19.6	23.2	0.002
Alcohol (%)				
Daily	44.3	34.1	22.8	< 0.001
Sometimes	12.9	12.9	9.5	
None/rarely	42.8	53.0	67.7	
Smoking (%)				
Current	14.8	8.0	4.9	< 0.001
Former	40.0	31.0	21.9	
Never	45.2	61.0	73.3	
Self-perceived chewing ability (%)				
Can chew anything/most things	96.1	97.8	98.5	0.012
Do not chew much	3.9	2.2	1.5	
Frequency of going out (%)				
More than once a day	83.2	82.3	82.6	0.809
Less than once a day	16.8	17.7	17.4	
Medical history (%)				
Hypertension	46.2	47.8	44.5	0.581
Diabetes	10.3	11.6	13.6	0.089
Cancer	12.0	12.9	12.5	0.781
Stroke	6.4	6.9	5.0	0.371
Heart disease	17.2	14.0	12.3	0.024
COPD	3.4	3.9	3.2	0.868
BMI (kg/m^2^)	23.3 (3.0)	22.9 (2.9)	22.8 (3.2)	0.008
Energy intake (kcal/day)	2033 (535)	2019 (532)	1918 (469)	< 0.001
MMSE (score)	28.4 (1.8)	28.5 (1.7)	28.6 (1.6)	0.009

*Abbreviations*: *COPD* chronic obstructive pulmonary disease, *BMI* body mass index, *MMSE* Mini-Mental State Examination; Data are means (SD) or percentages; *P values are based on the weighted one-way analysis of variance, for continuous variables, and the Mantel–Haenszel chi-square, for categorical variables

The prevalence of sarcopenia was 10.5%. The ORs for sarcopenia, according to tertile of dietary pattern 1 scores, are shown in Table 4. In the age-, sex-, and area-adjusted model (model 1), the first dietary pattern was significantly associated with decreased sarcopenia prevalence. After further adjustment for other covariates (model 2), the inverse association remained: the multivariable-adjusted odds ratio of sarcopenia was 0.57 (95% CI, 0.34–0.94; p for trend=0.022) in the highest versus the lowest tertile of dietary pattern.

**Table 4:** Odds ratios and 95% confidence intervals for sarcopenia, by tertile of dietary pattern 1 scores

	Dietary Pattern 1 scores			*p* for trend
	Tertile 1 (low)	Tertile 2	Tertile 3 (high)	
Subjects with/without sarcopenia	63/472	63/473	43/492	
Model 1^a^	1.00 (reference)	0.83 (0.55–1.25)	0.56 (0.35–0.88)	0.012
Model 2^b^	1.00 (reference)	0.88 (0.56–1.37)	0.57 (0.34–0.94)	0.022

^a^Model 1 was adjusted for sex (men or women), age (years, continuous), and study site (Hatoyama, Kusatsu, or Itabashi)

^b^Model 2 was adjusted for variables in Model 1 plus education (years, continuous), living alone (single, with spouse only, or other), smoking habits (current, former, or never), drinking habits (every day, sometimes, or none/rarely), self-perceived chewing ability (can chew anything/almost anything or cannot chew much), frequency of going out (more than once a day or less than once a day), medical history (hypertension, diabetes, cancer, stroke, heart disease, and chronic obstructive pulmonary disease; yes or no), body mass index (kg/m^2^, continuous), energy intake (kcal/d, continuous) and MMSE (score, continuous)

Table 5 shows associations of the first dietary pattern derived from RRR with components of sarcopenia. After adjustment for covariates, a dietary pattern characterized by high intakes of fish, soybean products, potatoes, most vegetables, mushrooms, seaweeds, and fruits and a low intake of rice was significantly positively associated with usual gait speed (β: 0.02, *p*=0.024). In contrast, RRR-derived dietary pattern was not significantly associated with ALM (β: 0.02, *p*=0.164) or grip strength (β: 0.31, *p*=0.059). Participants in the highest tertiles of the first dietary pattern score had greater ALM and grip strength and faster usual gait speed than did those in the lowest tertiles.

**Table 5:** Association between tertile of dietary pattern 1 scores and components of sarcopenia

	Dietary pattern 1 scores			Effects per one-tertile increase	*p* for trend
	Tertile 1	Tertile 2	Tertile 3		
	Mean (SE)				
Appendicular lean mass (kg/m^2^)					
Model 1^a^	6.5 (0.03)	6.5 (0.03)	6.6 (0.03)	0.01	0.701
Model 2^b^	6.5 (0.02)	6.5 (0.02)	6.6 (0.02)	0.02	0.164
Grip strength (kg)					
Model 1^a^	27.8 (0.2)	27.9 (0.2)	28.4 (0.2)	0.33	0.047
Model 2^b^	27.8 (0.2)	27.9 (0.2)	28.4 (0.2)	0.31	0.059
Usual gait speed (m/s)					
Model 1^a^	1.33 (0.01)	1.36 (0.01)	1.37 (0.01)	0.02	0.004
Model 2^b^	1.33 (0.01)	1.35 (0.01)	1.37 (0.01)	0.02	0.024

^a^Model 1 was adjusted for sex (men or women), age (years, continuous), and study site (Hatoyama, Kusatsu, or Itabashi)

^b^Model 2 was adjusted for variables in Model 1 plus education (years, continuous), living alone (single, with spouse only, or other), smoking habits (current, former, or never), drinking habits (every day, sometimes, or none/rarely), self-perceived chewing ability (can chew anything/almost anything or cannot chew much), frequency of going out (more than once a day or less than once a day), medical history (hypertension, diabetes, cancer, stroke, heart disease, and chronic obstructive pulmonary disease; yes or no), body mass index (kg/m^2^, continuous), energy intake (kcal/d, continuous) and MMSE (score, continuous)

## Discussion

In this cross-sectional study, we identified a dietary pattern associated with lower sarcopenia prevalence and improvement in sarcopenia components in community-dwelling older Japanese. This dietary pattern was characterized by high intakes of fish, soybean products, potatoes, most vegetables, mushrooms, seaweeds, and fruits and a low intake of rice. To our knowledge, this is the first study to use RRR analysis to identify dietary pattern and examine the association of this pattern with sarcopenia and its components.

In this study, the RRR-derived dietary pattern was inversely associated with sarcopenia among community-dwelling older adults. A recent review summarized the evidence from observational studies that used a priori methods (e.g., diet quality scores or indexes) and a posteriori approaches (e.g., factor analysis including PCA) to identify dietary patterns and examine the relationships of these patterns with sarcopenia and its components [7]. As compared with other age-related conditions, sarcopenia is less likely to be investigated as an outcome; however, four studies examined the association between dietary patterns and sarcopenia [39–42]. In a prospective study of community-dwelling older Chinese, a higher diet quality index (DQI-I) score was associated with reduced risk of prevalent sarcopenia in men but not in women [39]. Another study reported that a higher Baltic sea diet score (dietary index developed to account for “beneficial” foods consumed routinely in Nordic countries, such as berries, salmon, rapeseed oil, and dairy) was associated with a lower risk of sarcopenia in older women over a 3-year follow-up period [40].

Among studies using a posteriori approaches, a cross-sectional study of Iranian older adults reported that a Mediterranean diet (MED)-style dietary pattern derived by using PCA (with high factor loading for MED foods such as olives/olive oil, fruits, vegetables, nuts, whole grains, and fish) was associated with reduced risk of prevalent sarcopenia [41]. Among community-dwelling older adults from the Newcastle 85+ Study, a dietary pattern characteristic of a traditional British diet, as identified by cluster analysis (high intakes of butter, red meat, gravy, potato, vegetables, and sweets/desserts), was associated with increased sarcopenia risk [42]. Given the limited evidence regarding the association between dietary pattern and sarcopenia, however, further investigation is warranted.

The pattern we identified was positively associated with fish, soybean products, potatoes, most vegetables, mushrooms, seaweeds and fruits. In a recent review of studies of the relationship between individual whole foods and muscle health, a higher intake of fruits and vegetables was beneficial for muscle function in observational studies [43]. Although there was limited or inconclusive evidence for other whole foods (e.g., fish and soy products) in relation to sarcopenia and muscle-related outcomes [43], a combination of these food groups may contribute to nutrient adequacy for a range of nutrients for sarcopenia and muscle-related outcomes in older adults. Interestingly, our results showed that the RRR-derived dietary pattern was negatively associated with rice. Although rice is a large part of the Japanese daily diet, results similar to those of the present study have been reported in previous studies of dietary pattern in Japanese populations [32, 38, 44]. Ozawa et al. suggested that this association may be attributable to an imbalance in food intake (ie, a high rice intake may result in lower intakes of foods favorable for sarcopenia) rather than to any direct harmful effects of rice [38]. Indeed, rice intake was not significantly associated with sarcopenia in our supplementary analysis (data not shown).

In the present study, an RRR-derived dietary pattern was significantly positively associated with usual gait speed, but the association between RRR-derived dietary pattern and ALM was weak. In line with our findings, a previous systematic review reported that current observational evidence of a positive relationship between diet quality and physical performance is strong, whereas the evidence for an association between diet quality and muscle mass is weak [6]. Another review reported that the most consistent results were observed for the association of MED-type dietary patterns with physical functioning (lower extremity functioning, mobility, walking speed); the results were inconclusive for the association of dietary pattern with other components of sarcopenia, including muscle mass and strength [7]. These findings suggest that diet alone cannot offset age-related decline in muscle mass and that a combination of physical activity and adequate nutrition are more important for muscle mass. Alternatively, as the present dietary pattern shared characteristics with healthy dietary patterns characterized by higher intakes of beneficial foods (e.g., fruits and vegetables, fish), it may be necessary to consider other aspects of a healthy diet when designing an optimal diet for prevention of age-related decline in muscle mass (reviewed in Granic et al. [7]). Future studies should examine the effects of dietary pattern on muscle mass and the interaction of diet and physical activity.

The strengths of the present study include its use of data combined from three cohorts in Japan, the use of RRR to identify dietary patterns, utilization of a validated dietary questionnaire, and use of Asian-specific criteria to define sarcopenia. Our study also had several limitations that warrant mention. First, as this study is cross-sectional in design, we could not assess causality. Second, despite adjustment, we cannot completely rule out the effects of residual confounding by unmeasured or unstandardized variables on the association between the identified dietary pattern and sarcopenia. For example, because we were unable to obtain standardized variables across the cohorts, we could not adjust for physical activity and depression. Third, this study included relatively well-functioning community-dwelling elders who were able to participate independently in evaluations at community centers, which may have caused some selection bias. Fourth, we lacked data on use of dietary supplements. Finally, because RRR analysis depends on the data at hand, the findings are not entirely reproducible by other studies. Therefore, replication in other cohorts is needed in order to confirm the generalizability of our findings. Moreover, although the present study areas included an urban area, a suburban area, and a rural area, most are located in eastern Japan (Kanto region). Thus, studies of persons living in other areas of Japan are warranted in order to confirm the generalizability of the findings.

## Conclusions

In conclusion, a dietary pattern characterized by high intakes of fish, soybean products, potatoes, most vegetables, mushrooms, seaweeds, and fruits and a low intake of rice was significantly inversely associated with sarcopenia. Our findings suggest that sarcopenia can be delayed or prevented by improving diet quality. However, further research, especially prospective studies, is necessary in order to confirm this hypothesis.

## Supplementary Information

**Additional file 1.** STROBE-nut: An extension of the STROBE statement for nutritional epidemiology.

## Data Availability

The datasets used and analyzed in this study are available from the corresponding author on reasonable request.

## Abbreviations

RRR: Reduced rank regression
PCA: Principal component analysis
ALM: Appendicular lean mass
BDHQ: Brief self-administered diet history questionnaire
COPD: Chronic obstructive pulmonary disease
BMI: Body mass index
OR: Odds ratio
CI: Confidence interval
DQI-I: Diet quality index
MED: Mediterranean diet
